# Balancing Objectivity and Humanity: Ethical Challenges and Considerations in Surgical Candidacy Decisions

**DOI:** 10.1007/s10730-025-09565-6

**Published:** 2025-08-31

**Authors:** Anthony S. Peterson, Bryan Pilkington

**Affiliations:** Department of Medical Science, Hackensack Meridian School of Medicine, 123 Metro Boulevard, Nutley, NJ 07110 USA

**Keywords:** Surgery, Candidacy, Surgical risk, Risk assessment, Human factors

## Abstract

An essential element of determining surgical candidacy is an accurate understanding of the risks to a given patient. While surgeons remain largely responsible for the selection of their patients, and surgeons’ intuition has been shown to be a good indicator of postoperative outcomes, the recent focus in medicine towards minimizing the impact of physician bias has spurred a push towards prioritizing risk assessment tools in candidacy decisions. This has rekindled the debate surrounding what should determine surgical candidacy. Risk assessment tools are proven to be moderately to highly accurate at assessing the risk due to objective and proven risk factors, such as the impact of age or comorbidities. However, they fail to account for the humanity of both the surgeon and the patient and do not measure less easily quantifiable risk factors, such as a surgeon’s comfort with a procedure or a patient’s health beliefs, when determining risk. In this project, we offer an ethical analysis that highlights these less acknowledged factors. We argue that these factors need to be given greater consideration in risk assessment and surgical candidacy decisions, as they too can affect postoperative risks and outcomes.

## Introduction

A foundational dilemma in medicine has always been who to treat. No treatment comes without risks or potential harm to the patient, and so an accurate understanding of all^1^ risks and benefits is necessary to decide when and how to treat. As technology has advanced, surgery has become a standard treatment for an increasing number of medical conditions but carries with it very real risks. Historically, surgeons, due to their familiarity with both the procedure and the patient, were solely responsible for determining if their patients were proper candidates for surgery. It fell within their purview to assess whether the benefits of a given surgery were worth the risks to the patient. Beyond simply mastering the technical side of the craft, the art of surgery was in knowing when to cut, and when not to. This mindset is well encapsulated in the old adage: “Good surgeons know how to operate, better ones when to operate, and the best when not to operate” (BMJ, 1999).

As decision-making within medicine has moved from a paternalistic to a patient-led model, concerns have been raised as to how accurately surgeons are able to estimate the risk to a patient from a given surgery, as a surgeon’s decision-making can be clouded by various cognitive biases—such as overconfidence, anchoring, and confirmation biases—that can lead to patient harm (Armstrong et al., 2023; Jacklin et al., 2009). Everything from the surgeon’s mood to their relationship with the patient could, in theory, impact their decision about whether or not to perform surgery. These concerns have led to the creation of risk assessment tools (RATs), such as the American College of Surgeon’s National Surgical Quality Improvement Program Surgical Risk Calculator (ACS NSQIP, 2023), with the aim of eliminating these subjective factors and therefore more objectively, and ideally more accurately, estimating surgical risks (Bilimoria et al., 2013; Bronsert et al., 2020).

As various RATs have been developed, validated,^2^ and improved, however, they have failed to consistently outperform surgeons’ risk assessments, and questions remain as to why (Dilaver et al., 2020; Huckaby et al., 2022). These unexpected findings suggest that there may be other variables not currently under consideration that likely play into surgical candidacy decisions—an issue that is only exacerbated by the apparent lack of consensus as to what makes a patient a “fit” candidate for surgery.

In this paper, we seek to present a more cohesive narrative of the various factors—surgeon-related, patient-related, and other—that are integral to truly accurate risk assessment calculations and better informed surgical candidacy decisions. We do this by first examining why neither RATs nor surgeon decision-making are sufficiently accurate for candidacy decisions through the lens of some oft-presented but false assumptions that are included in these discussions. We then reframe these assumptions by presenting data that highlights many non-surgeon, non-patient factors that have been shown to play into candidacy decisions but are currently not included in the decision-making. Finally, we provide an example of what this holistic approach towards surgical candidacy decisions that takes into account all relevant factors could look like.

## A Problem of Consistency

Consider first a common anecdote in discussions of surgical candidacy: a single patient visits two different surgeons to discuss getting a specific procedure. Both surgeons use the same RAT to assess the patient and get the same risk score, but one surgeon declines to offer surgery and the other offers it. There are many possible reasons why both surgeons did not reach the same decision, whether that be scheduling concerns, risk of litigation, willingness to take on higher-risk patients, skill level concerns, or any number of other factors. However, if an objective calculation of risk based on measurable risk factors was the sole driver of candidacy, both surgeons should have agreed (or disagreed) to the procedure. Instead, this scenario that regularly plays out—especially with higher-risk patients—suggests that there are other variables being considered in these decisions. An understanding of what variables are relied upon in risk assessment is therefore key to better knowing when to cut and when not to.

This work carries with it additional urgency in the age of artificial intelligence (AI). Although a full discussion of this is beyond the scope of this paper, the capabilities of AI will likely be essential to developing truly accurate RATs, as algorithms that can tailor risk to the individual promise to both improve risk assessment accuracy as well as help surgeons in avoiding bias, but can only do so if they are designed in the right ways (Binkley et al., 2022a, 2022b). This, however, requires first and foremost understanding of what changes must be made. Additionally, although candidacy for surgery and debates over risk assessments are not new subjects in surgical ethics, one further consideration—which we take up below—that has received insufficient treatment, is the differing skill sets of surgeons, suggesting that there may be no single objective assessment of surgical candidacy, but rather that candidacy can only be understood in relation to time, place, and the particularity of the physician who might perform the procedure or, better, of the physician–patient dyad under consideration.

If this is correct, it complicates the view of surgical candidacy in a variety of ways, raising additional ethical issues. It highlights a *human* resource that has not been attended to sufficiently in resource allocation discussions, for example. If it is true that surgical candidacy is relational, then increasing access to surgery would not sufficiently address the issue; rather, access to surgeons willing and able to perform the relevant surgery would be needed. This is, of course, a high bar, but if we are to take access (Khubchandani et al., 2018) and equity (Rabbitts & Groenewald, 2020) concerns seriously, it is one that must be engaged. A second ethical issue that needs to be highlighted, and, again, the bar is high, is that surgeons must be honest with themselves, their patients, and society. This means frank assessments of their own skills, the willingness to be reviewed by colleagues, and the openness with patients to say they may or may not have the skills to offer all of the surgical options that others might. This issue will be discussed further, but before reflecting on these possibilities, we focus on possible solutions to disagreements about candidacy. These solutions, if viable, could supersede the need to find immediate answers to the more challenging ethical issues of resource allocation and access and critical self-reflection.

## Potential Responses to the Problem

One response to disagreements over candidacy, as realized through cases like the aforementioned, is simply to use both RATs and the surgeons’ intuition together in formulating the final decision, potentially bringing together sources of knowledge that might cover all bases. RATs eliminate the cognitive biases surgeons can be subject to but are limited in the variables they can measure. Surgeons are potentially susceptible to biases but can more readily take into account subjective risk factors as well as technical considerations such as anatomy. Bringing them together theoretically allows for the best possible surgical decision by maximizing the benefits of each. However, due to concerns about the current failings of RATs—namely their inconsistent superiority to surgeon judgement—the majority of surgeons have not integrated them into their practice (Eamer et al., 2018; Keller et al., 2018; Pradhan et al., 2022).

Interestingly, perhaps the largest systematic review to date comparing the accuracy of surgeons’ risk assessments versus those of RATs found that surgeons generally overestimate, not underestimate, the risk of mortality (Divaler et al.,^ 2020^). The fact that surgeons tend to consistently overestimate risk could suggest a protective element^3^ in doing so, as opposed to an inaccurate understanding as to the risk presented by a given procedure. This could further explain the hesitancy to adopt the usage of RATs into general practice. Furthermore, another study found that even though the use of RAT data improved risk assessment accuracy, it did not change the likelihood of the surgeon recommending an operation (Sacks et al., 2016). Could this be because it does not take into account the particular skill set and surgical preferences of individual surgeons? Or could it have to do with an understanding that RATs only look at a few of many factors that impact surgical outcomes? We do not have data to support or deny these conjectures. However, we do know that multiple studies have concluded that the best use of RAT data seems to simply be for use in explaining and illustrating the risks to the patient by showing them visually, in real time, how changes such as smoking cessation affect the overall risk calculation (Chand et al., 2007; Huckaby et al., 2022).

Perhaps these findings alone are enough to warrant the use of RATs, in the name of informed consent, which is a vital part of protecting patient autonomy when done properly. Some studies have found that the use of RATs in the consent process improves patient satisfaction and reduces anxiety around the risks (Raymond et al., 2019; Wiesen et al., 2020), furthering the argument for the inclusion of RATs in these discussions. However, although there is merit to these arguments, similar studies have found that any aide—whether a visual graphic or written document explaining the risks—has the same effect, which once again negates the absolute need for RATs, especially as they are now (Jalal et al., 2024). Regardless, the focus of these arguments is on the accuracy of RATs, which as we have already shown, are insufficient as they are.

What we are getting at is that, interestingly, in none of these conversations—as opposed to conversations surrounding medical error—is consideration given to the human factors, or the more subjective risk factors that affect surgical outcomes. These risk factors arise from the humanity of both the surgeon and the patient and are unique to each patient-surgeon dyad. Moreover, these human factors may both account for, and explain, the consistent inaccuracies in RAT data, as well as explain why candidacy decisions can be so complicated and difficult. Surgeons are not, as no group of professionals are, identically skilled. This leads to our aforementioned ethical concerns around critical self-reflection and honesty, as well as resource allocation and access. If surgeons have widely ranging skill sets, widely varying comfort levels with different procedures, and different propensities for risk-taking, how then are members of patient populations in need of surgical care to understand what might be consistently offered? Or, put in another way, how can surgeons offer reasonable expectations to patients when skill, at least in theory, plays such a critical role in outcomes and complication rates?^4^ To put this in numbers, although the vast majority of complications are minor, the absolute surgical complication rate in the US has been estimated between 7 and 15% for patients undergoing major surgery (Dencker et al., 2021). When looking at specific specialties or surgeries that estimate ranges anywhere from 2 to 30% or more, largely based on the complexity and inherent risks of the procedure(s) performed (Birkmeyer et al., 2013; Dencker et al., 2021; Healey et al., 2002). However, assuming even just a 7% complication rate, this equates to upwards of millions of surgical complications a year.

In terms of surgeon-specific complications, the current consensus in medical error literature is that the surgeon’s skill may account for up to roughly 25% of the variation in surgical outcomes (Stulberg et al., 2020). How accurate that value is continues to be debated and researched, but assuming it is reasonably accurate this means that each year hundreds of thousands of complications could be attributed in some way to technical skill. The ideal, of course, is to have as few complications as possible and for no unnecessary risk to be added to a surgery for any reason. However, as long as humans are performing the surgeries, some element of error related to natural human imperfections must by necessity be present. That has always been an accepted and expected risk of undergoing surgery.

With that said, these complications, even if attributable to skill [and one could argue that many of the errors come not directly as a result of the surgeons’ skill but instead due to institutional and procedural factors such as exhaustion from being overworked, pressure to make surgeries as short as possible, and/or lack of adequate resources (Gunaratnam & Bernstein, 2018)], should not serve as a condemnation of surgeons, the vast majority of whom are striving to continually hone and improve their craft^5^ and have their patients’ best interests in mind. Along this vein, Gunaratnam and Bernstein (2018) found that most surgeons self-reported including factors such as their comfort with a specific procedure and the availability of resources in their candidacy decisions, and reported a frank willingness to refer their patients to experts with better outcomes for specialized procedures when possible.^6^ Assuming this is indeed true, it strongly suggests that surgeons are already considering their own role in patient outcomes. Perhaps, then, it is factors other than the surgeon and the patient that are accounting for the inconsistencies with surgical candidacy decisions. Instead, as will be discussed, we believe that there are many other factors that could, if properly addressed, potentially lower complication rates significantly more than any intervention focused on technical skill could.

As a final note: in relation to surgical skill, it is important to recognize that while there is significant disagreement as to what extent surgical skill actually does directly affect outcomes, one major consensus seems to be that while lower skill levels are associated with increased short-term complications, in the long term outcomes do not seem to be solely attributable to skill level (Birkmeyer et al., 2013; Scally et al., 2016; Varban et al., 2021). In other words, while two surgeons of varying skill will both achieve the desired surgical outcome, such as successful repair of a hernia, short-term complications such as infections or damage to nearby structures will be more common with the less-skilled surgeon, likely due to factors such as longer operating time, less proficiency with the instruments, and less familiarity with operating room procedures. However, the most common reason for “lower skill” is less experience, and so these findings might just be an unavoidable part of the training process, and are already an accepted part of that.^7^ Thus, while efforts to lower complications rates by focusing on surgical skill will always be worthwhile, especially for reducing short-term complications and costs, other factors that may contribute to complications must also be considered, as targeting them might have a greater total possible impact on lowering complications.

While the discussion thus far has focused on the potential impact of surgeons’ skill on surgical complications, within any surgery there is by necessity another major player: the patient. If, hypothetically, we were to assume that the surgeon and the patient were truly the only two players in a surgery—aka the “surgeon–patient dyad”, a seemingly fair (albeit false) assumption, as RATs only account for the patient factors, medical error literature focuses mostly on surgical skill, and few other factors are ever discussed—this means that the patient factors included on RATs must account for the other 75% of variation in complications that cannot be explained by surgical skill. If that were the case, however, should not more accurate and robust RATs have long since been developed? In addition, would not simply adding a section on RATs to factor in surgeons’ skill then be enough to fix the RATs’ failings? Perhaps. On the other hand, as suggested by Gunaratnam and Bernstein (2018), are not the vast majority of surgeons already factoring their skill into candidacy decisions? This would seem likely. Instead, perhaps “surgeon error” is simply the easiest explanation for why things go wrong, when in reality it is only a small piece of a larger network of factors that antecede surgical complications.

Before continuing further, something must be said about the implications of complications in improving medical knowledge. Although the end goal is, of course, for surgical complications to eventually be eliminated, they currently play an important role in developing and improving surgical techniques and devices. Often complication reduction is an iterative process: attempts at complication reduction lead to better knowledge, which leads to better outcomes. For example, many of the improvements in reverse total shoulder arthroplasty came after complications such as scapular notching and scapular spine fracture arose and were then studied (Frankle et al., 2005; Larose et al., 2022). Subsequent improvements made based on that research have both reduced the rate of those complications and allowed for better overall outcomes for all patients. There also continue to be many surgeries for which factors that affect outcomes are still being discovered. With rotator cuff repairs, for example, a better understanding of fatty changes has allowed for the establishment of better surgical candidacy criteria because higher-grade fatty changes are now known to be a significant predictor of surgical failure (Baek et al., 2022; Osti et al., 2014). Today, when patients present with high-grade fatty changes, alternative surgical options are often pursued to bring better outcomes. Thus, it seems that even complications are not cut and dry. There are, of course, some complications that arise solely from technical error, but many also arise simply by chance or because of deficiencies in medical knowledge. This also highlights the necessity of establishing non-punitive ways to look at surgical complications. If policies are implemented in such a way that complications are subsequently hidden or brushed aside to avoid undue punishment, the consequences would be detrimental to medicine as a whole. One other conclusion that can be drawn is that as knowledge improves, so do outcomes, which illustrates exactly why additional factors that could affect surgical outcomes must be studied, understood, and addressed.

A first, and obvious, step in better understanding what these other factors that can impact surgical risk, and thus surgical candidacy, are, is to investigate what those factors might be. Within the recent literature on the subject, there are a number of potential risk factors, completely unrelated to technical skill and medical knowledge, that have been investigated and that have been shown to impact surgical outcomes to various degrees. The wide-ranging nature of these factors serves to elucidate the amount of work that must be done to develop a comprehensive understanding of surgical risk. The first set of these risks relate specifically to the surgeon. These risk factors include, but are not limited to, personality and leadership style (Bisset et al., 2020; Contessa et al., 2013; Shubeck et al., 2019), practice location and volume load (rural surgeons, by necessity, must be jacks of all trades, whereas surgeons in more urban centers are able to master just a few main operations; Shaydakov & Tuma, 2024), and the surgeon’s nontechnical skills (such as the ability to cope with intraoperative stressors; Arora et al., 2010; Hull et al., 2012). These factors reveal many possible interventions that could be implemented to improve long-term surgical outcomes. For example, if the findings of Shubeck et al. (2019) are correct, certain leadership skills are directly correlated with decreased rates of adverse surgical outcomes, which suggests a need to develop training to cultivate those traits in surgeons. Along a slightly different vein, Arora et al. (2010) found that higher intraoperative stressors lead to worse surgical outcomes. Interestingly, they concluded that the high stress levels brought about a subsequent deterioration in technical skills, which then negatively impacted outcomes. This once again suggests that perhaps it is not the actual skill of the surgeon in a vacuum, but other factors—such as excess pressures placed on surgeons—that are the major drivers of complications related to technical skill.^8^

Along with surgeon-specific factors, such as those discussed, there exist some patient health behaviors and features of the patient’s situation that can also impact the downstream outcome of a procedure. These factors include the patient’s access to healthy food (Chinaemelum et al., 2023; Tolkacz et al., 2024), religious beliefs (Contrada et al., 2004; Coruh et al., 2005), and mental health (Laurino Neto & Herbella, 2019). Health behaviors such as smoking and patient characteristics such as BMI have long been included in surgical candidacy decisions due to their known and measured impact on surgical outcomes. However, it is also widely known that nutrition is an integral part of health, from which it naturally follows that poor nutrition should also directly correlate with poor surgical outcomes, as found by Chinaemelum et al. (2023) and Tolkacz et al. (2024). Why, then, are nutrition and/or food insecurity not included on RATs? The most likely reason is that research has not yet been able to quantify the exact impact of nutrition and food insecurity on surgical complication rate—a much more nuanced and complex challenge than measuring the impact of more readily defined factors such as age—which shows yet another difficulty with developing accurate estimates of surgical risk.

This difficulty is only exacerbated by the subjective and individualistic nature of many of these risk factors. For example, within the realm of religion and spirituality, there exist thousands of different religions worldwide, each with different beliefs and teachings. Many of these beliefs—such as whether one can receive a blood transfusion, God versus the physician’s role in healing, and the utilization of alternative or religious-based therapies in the healing process, can all affect outcomes. However, quantifying the impact of each unique set of religious beliefs and practices, especially since there is wide variance in how each religion is practiced on the individual level, on surgical outcomes would be nearly impossible. For mental health factors, many of the concerns are the same: in light of mental health struggles, how well will the patient be able to cope with the stresses of surgery and the life changes that result, and how will their illness impact their healing journey. Perhaps more importantly, the wide-ranging nature of these patient-related factors is beyond the scope of what any RAT would currently be able to quantify and handle, and how much benefit could be derived from doing so is as yet unknown. Instead, our discussion of these factors simply serves to demonstrate just how complicated accurately and objectively determining surgical risk can be.

Looking beyond the surgeon and the patient, matters are further complicated when more abstract risk factors are taken into account. These factors readily illustrate that the above assumption—that the only two players in a surgery are the surgeon and the patient—is far from the truth. That conclusion alone largely invalidates the current focus of RATs and accepted risk assessment methods. Put in another way, even if patient factors account for 50% of surgical risk (double that attributable to surgeon skill), there is still 25% of risk left unaccounted for. It is more than likely that these abstract risk factors make up this remaining portion of surgical risk, making them a significant contributor to surgical outcomes. One example of these risk factors is the weather. There is substantial literature showing that the weather and season can significantly impact various surgical outcomes. Warmer weather and increased humidity at the time of surgery are correlated with more surgical site infections (one of the most common surgical complications) (Gautam et al., 2011; Sahtoe et al., 2021), while winter is associated with worsened overall surgical outcomes (Spencer et al., 2022). These seasonal variations are not currently included in RAT calculations, even though many readily include the risk of getting a surgical site infection (Scarborough et al., 2016). The time of day the surgery takes place also seems to have some impact on outcomes. In general, daytime-hour surgeries tend to have significantly lower complication rates than off-hour surgeries (Cortegiani et al., 2019; Halvachizadeh et al., 2019; Phatak et al., 2014). For most surgeries, morning slots seem to lead to better outcomes than afternoon slots (Ishiyama et al., 2019). For others, the reverse is true (Montaigne et al., 2018). Still, for others, the time of day does not seem to matter (Sessler et al., 2011). Once again, these considerations are not included on RATs or in surgical scheduling decisions, and doing so would be highly complicated, if not impossible. A final few currently researched risk factors include the surgeons’ familiarity with the surgical team (Kurmann et al., 2014), number of intraoperative distractions (Mentis et al., 2016; Wheelock et al., 2015), and whether the patient has a recovery room with a window (Wang et al., 2019).

To give the best possible risk assessment, should not the added risk from each of these factors be included in risk calculations as much as patient comorbidities?^9^ While the answer seems clear, the application is not. Namely, if our suggestion to account for more risk factors (especially, but not only, those human factors connected to the physician–patient dyad) is taken seriously, these additional risk factors must be included in future RATs and risk assessment schemata. However, the sheer variety and volume of these human factors readily illustrates just how impossible accounting for every single one would be. Additionally, this data makes clear that where researched statistics and objective risk measures can predict population-level risks, they cannot perfectly translate down to the individual level (Chand et al., 2007). There is simply too much variation between individuals to lump them into such fixed categories. Therefore, in order to provide a much more realistic risk score, a truly accurate RAT would have to provide a number completely unique to the individual patient, not a pre-calculated value, and that number would have to take into account the time, place, and particulars of each surgeon–patient dyad. We envision the eventual creation of a system by which patients are matched with surgeons based on the specific issues and needs of the patient, therefore minimizing overall risk system-wide. However, until then, the first step is designing and implementing more accurate RATS.

Much more work needs to be done to figure out how to best design and implement a more accurate and cohesive RAT. However, based on what we have presented above, one way this could be achieved would be to expand current RATs into three sections^10^: (1) patient factors, (2) surgeon factors, and (3) hospital/procedural/other factors. The first section would look at both objective and social patient factors—both those currently included comorbidities such as diabetes and hypertension as well as social factors such as smoking status, access to nutrition, and ability to participate in rehab/physical therapy. The second section would include some metric of surgeon skills, such as the surgeon’s complication rates for the given surgery compared to a specialty-wide standard.^11^ The third section would do the same for the hospital as a whole (i.e. the overall complication rates of the hospital compared to a nation-wide standard) as well as include any other risk factors not included elsewhere. How exactly this sort of framework is designed and implemented will likely depend on further developments with the field of AI, as well as how the conversation on surgical candidacy evolves, and is beyond the scope of this paper. However, more work on these issues is essential, as surgeons have an ethical responsibility to fully inform patients not just about the risks of surgery due to the patient’s own comorbidities and risk factors^12^ but also the risks related to the surgeon and facility in which they will undergo the procedure. However, this can only be achieved if further work is put in to determining what truly makes an individual a candidate for surgery and what factors impact the risk of the surgery, because, as we have shown, thus far no objective criteria have been able to clearly and accurately delineate those “fit” from surgery from those “unfit” for surgery.

## Conclusion

There is currently no clear standard as to what amount of risk should disqualify a patient from surgery. However, no standard can be developed until these aforementioned human factors are given more focus and attention. These are hard conversations to have, as they bring to the forefront considerations of hospital policies and resources as well as colleagues' skill sets relative to those of others and highlight further access inequities that are outside of the control of patients—one might simply live in an area with fewer (or less skilled) surgeons or fewer available resources. As medicine continues to advance and more research is published on objective and measurable risk factors, both RATs and physicians’ judgments will continue to get more accurate, to a point, but some element of surgical outcomes will always come down to the multitude of factors that can play into outcomes, such as the individuality of each patient, surgeon, place, and time. Ethically, we must be clear, consistent, and open about this fact, and this means including elements addressed above in the informed consent process for surgeries if surgeons, in particular, and health systems, more generally, are to respect the dignity and autonomy of patients.

That said, surgeons may currently be in a better situation than our arguments have led the reader to understand. As studies have shown, surgeons’ gut feelings are often accurate with regard to surgical outcomes (Hartley & Sagar, 1994; Markus et al., 2005). Interestingly, this seems to be an accepted view, as many RATs include a spot for the surgeon to alter the output based on their intuitions regarding the patient’s health status. Those gut feelings perhaps reflect an innate and subconscious understanding regarding the impact of these human factors, or so we suppose. Additionally, one study in 2009 found that providing cognitive feedback improved surgeons’ risk estimation reliability (Jacklin et al.,^ 2009^). Unfortunately, in the wake of RATs, not much follow-up has been done on those findings. Perhaps along with continuing to develop and improve RATs, training could be developed to help surgeons better understand and account for the human factors that technology cannot. On the other hand, perhaps there can be no perfect standard developed to readily determine surgical candidacy because there is no objective answer as to what makes a good surgical candidate, and therefore measuring and quantifying known risk factors is the best that can be done in making accurate candidacy decisions.

When such measuring occurs, three things are of the utmost importance. First, surgeons must be honest with themselves and with their patients, not just about their estimates of success relative to the patient’s values but also about their own skill set—that is, their own ability to produce those results. They must, of course, take into account the other factors highlighted above that suggest against thinking of surgical candidacy as objective, but critical self-reflection continues to be needed to ensure that patient-specific factors are not the only ones addressed. Second, systems must be created to ensure that surgeons are given the best chance of success, such as through minimizing intraoperative distractions and finding ways to combat physician stress. Finally, as a society, we need to take seriously the varying skill sets of practitioners and try to put into place mechanisms for fair distribution of those *human* resources. Attending to human factors and to individual human capabilities—in a non-punitive way—may allow us to get ahead of potential mistakes and, with the right matching, increase access to needed surgery by creating better fits and better relationships among surgeon–patient dyads.

## Footnotes

1 Here we are referring to all plausibly known risks, not thinking of everything that is possible.

2 By validated we mean that the RATs were proven to be accurate enough to meet a standard, which has no comparative bearing on surgeon performance.

3 As far as we are aware, the motivations behind this have not been clarified in the literature, but they likely stem from a variety of influences, such as wanting to avoid unnecessary harm to patients as well as avoiding litigation.

4 We recognize that merely raising such provocative questions without answering them may leave the reader with concerns about the overall project in light of the pervasive nature of reasonable expectations in how patients seek care and how health systems provide care. A full answer to the question is outside of the scope of this paper, though we explore it elsewhere in more detail. In brief, we believe that surgeons must be honest about the varying skill sets within their specialty, that is, of themselves and their colleagues. Additionally, as they train and credential other surgeons, the minimum bar for succeeding in those spaces must be made clear. Finally, it is possible, given the exponential growth in both the power of AI and, more importantly, its influence on and involvement in health care delivery, that there may be individualized metrics which allow for greater and more accurate information to be shared with patients, relative to their individual situation and the anticipated minimal skill set of the surgeon needed to address their situation.

5 Aims to achieving excellence being a necessary condition for a profession (Buchanan, 2009).

6 This highlights that an emphasis on considerations of human factors in surgical procedures is not synonymous with a discussion of medical error; rather, it is to highlight the differences in skill between professionals. The fact that where the line is drawn—empirically or theoretically—results in some complications being attributed to error and others not is as interesting and important to us as distinctions between, say, exceptional and average care.

7 This is not to say that inexperience can be used as an excuse for lack of skill and worse outcomes. It is merely to emphasize that skill and experience can only be obtained from practice and time, meaning that some of these complications might be necessary to surgical training.

8 This further suggests that finding ways to lower surgeon stress should also lower complication rates. Those implications, however, are beyond the scope of this project.

9 To be clear, this same question should be applied to any other risk factors demonstrated in the literature.

10 Within which the included risk factors could then be adjusted and updated as future research better quantifies and delineates the most important risk factors for surgery.

11 This could alternatively/additionally include measures of technical skill, as work is currently being done to develop algorithms by which to measure and rate surgical skill (Lavanchy et al., 2021; Shafiei et al., 2023).

12 And therefore also give the patient the opportunity to lower the risks (such as smoking cessation or working towards better control of blood sugars) where possible prior to surgery.
